# Unveiling potential drug targets for hyperparathyroidism through genetic insights via Mendelian randomization and colocalization analyses

**DOI:** 10.1038/s41598-024-57100-3

**Published:** 2024-03-18

**Authors:** Bohong Chen, Lihui Wang, Shengyu Pu, Li Guo, Na Chai, Xinyue Sun, Xiaojiang Tang, Yu Ren, Jianjun He, Na Hao

**Affiliations:** 1 Department of Urology, The First Affiliated Hospital of Xi’an Jiaotong University, Xi’an, 710061 Shaan’xi Province China; 2 Department of Obstetrics and Gynecology, the First Affiliated Hospital of Xi’an Jiaotong University, Xi’an, 710061 Shaan’xi Province China; 3 Department of Breast Surgery, First Affiliated Hospital, School of Medicine, Xi’an Jiaotong University, 277 Yanta Western Rd., Xi’an 710061, Shaan’xi Province China; 4 Department of Neurology, The First Affiliated Hospital of Xi’an Jiaotong University, Xi’an, 710061 Shaan’xi Province China

**Keywords:** PIK3C3, SLC40A1, Hyperparathyroidism, Drug, Genetics, Mendelian randomization, Drug discovery, Endocrinology, Parathyroid diseases

## Abstract

Hyperparathyroidism (HPT) manifests as a complex condition with a substantial disease burden. While advances have been made in surgical interventions and non-surgical pharmacotherapy for the management of hyperparathyroidism, radical options to halt underlying disease progression remain lacking. Identifying putative genetic drivers and exploring novel drug targets that can impede HPT progression remain critical unmet needs. A Mendelian randomization (MR) analysis was performed to uncover putative therapeutic targets implicated in hyperparathyroidism pathology. Cis-expression quantitative trait loci (cis-eQTL) data serving as genetic instrumental variables were obtained from the eQTLGen Consortium and Genotype-Tissue Expression (GTEx) portal. Hyperparathyroidism summary statistics for single nucleotide polymorphism (SNP) associations were sourced from the FinnGen study (5590 cases; 361,988 controls). Colocalization analysis was performed to determine the probability of shared causal variants underlying SNP-hyperparathyroidism and SNP-eQTL links. Five drug targets (CMKLR1, FSTL1, IGSF11, PIK3C3 and SLC40A1) showed significant causation with hyperparathyroidism in both eQTLGen and GTEx cohorts by MR analysis. Specifically, phosphatidylinositol 3-kinase catalytic subunit type 3 (PIK3C3) and solute carrier family 40 member 1 (SLC40A1) showed strong evidence of colocalization with HPT. Multivariable MR and Phenome-Wide Association Study analyses indicated these two targets were not associated with other traits. Additionally, drug prediction analysis implies the potential of these two targets for future clinical applications. This study identifies PIK3C3 and SLC40A1 as potential genetically proxied druggable genes and promising therapeutic targets for hyperparathyroidism. Targeting PIK3C3 and SLC40A1 may offer effective novel pharmacotherapies for impeding hyperparathyroidism progression and reducing disease risk. These findings provide preliminary genetic insight into underlying drivers amenable to therapeutic manipulation, though further investigation is imperative to validate translational potential from preclinical models through clinical applications.

## Introduction

HPT arises from dysregulated parathyroid hormone (PTH) secretion, leading to disturbances in calcium and phosphorus homeostasis. It is primarily categorized into three types: primary, secondary, and tertiary hyperparathyroidism. Primary hyperparathyroidism (PHPT) represents the third most prevalent endocrine disorder worldwide. Over the past 50 years, the global incidence and prevalence of PHPT have notably increased. Notably, it is associated with parathyroid adenoma in more than 80% of cases. Secondary hyperparathyroidism (SHPT) arises from hypocalcemia, typically due to chronic kidney disease (CKD). CKD poses an escalating global health burden, with an estimated worldwide prevalence of 8–16%. Tertiary hyperparathyroidism occurs rarely by comparison, marked by autonomous and excessive parathyroid hormone secretion from hyperplastic glands triggered by prolonged SHPT^1^.

HPT is a complex and diverse condition that significantly impacts the quality of life for affected patients with various complications, including cardiovascular diseases^2^, kidney stones, and bone diseases^3^. In rare cases, prolonged hyperparathyroidism can ultimately induce end-stage parathyroid carcinoma—an aggressive malignancy with limited treatment options^4,5^. Along with the disorder of PTH, calcium, and phosphate levels, thorough evaluation and tight management are crucial for the treatment of HPT. The Fifth International Workshop guidelines provide a Grading of Recommendations, Assessment, Development, and Evaluation (GRADE) methodology to guide surgical management of asymptomatic primary hyperparathyroidism as well as non-surgical approaches for symptomatic cases^6^. However, these guidelines have only applied the GRADE approach to systematic review evidence rather than the process of formulating final recommendations. As a result, clinical practices continue to lack standardization, especially regarding the selection of pharmacological therapies for PHPT^7^. Furthermore, surgery is one of the recommendations for most primary hyperparathyroidism and tertiary hyperparathyroidism patients, but the long-term effect is poor and a substantial subgroup exists who are asymptomatic and may not derive significant benefits from surgery^8^. Therefore, it is essential to explore genetics and identify effective drug targets for the prevention of HPT.

While large-scale randomized controlled trials (RCTs) can rigorously assess pharmacological interventions, undertaking such studies requires extensive planning, prolonged execution timelines, and substantial resource allocation. Additionally, RCTs are not feasible to examine causal links between thousands of druggable genes and hyperparathyroidism in the absence of definitive evidence.

Recently, MR analysis has gained widespread usage in drug target development^9^. MR leverages the random assortment of genetic variants during conception to minimize confounding from environmental and lifestyle factors. Expression quantitative trait locis (eQTLs) linked to changes in gene expression may mimic long-term exposure to drugs targeting the encoded proteins^10,11^. Genome-Wide Association Study (GWAS) data on the outcome (e.g. hyperparathyroidism risk) can then be integrated with eQTL information to examine causal relationships between gene expression and disease using MR. Such approaches have been implemented for diseases like aortic aneurysms, COVID-19, rheumatoid arthritis, and Parkinson’s disease^12–15^. However, MR has yet to be applied to systematically evaluate possible treatment targets for hyperparathyroidism.

In this work, our objective was to identify potential drug targets related to HPT through MR analysis. In two separate exposure cohorts, we found a significant association between the genetically predicted expression of SLC40A1 and PIK3C3 and the risk of HPT, supported by robust evidence for colocalization. The result supports that drug-targeting PIK3C3 and SLC40A1 may slow down HPT progression, which offers a potential approach to prioritize medical treatment for the prevention of HPT.

## Methods

### Study design and data sources

**Figure 1:**
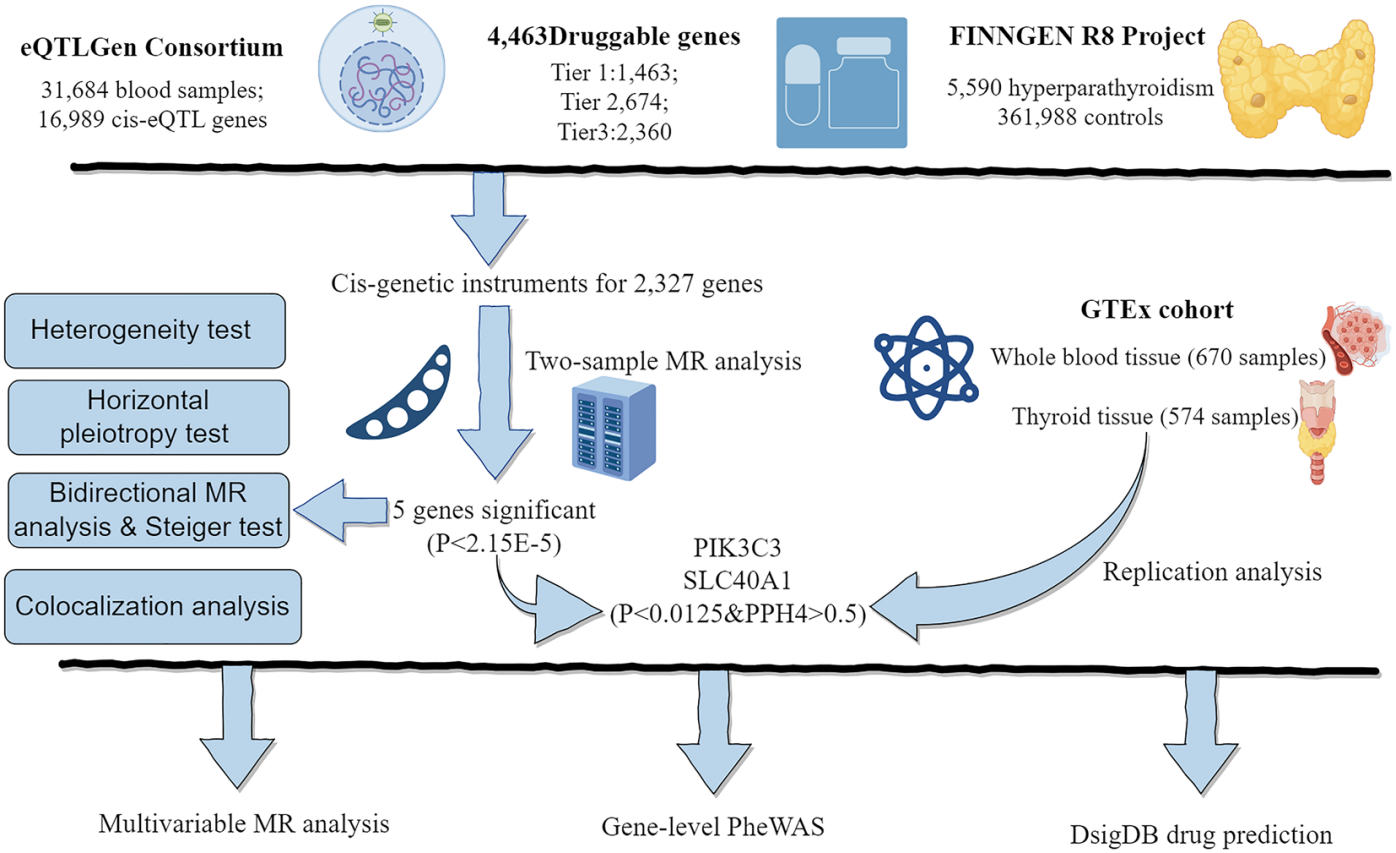
Study design overview (By Figdraw).

**Figure 2:**
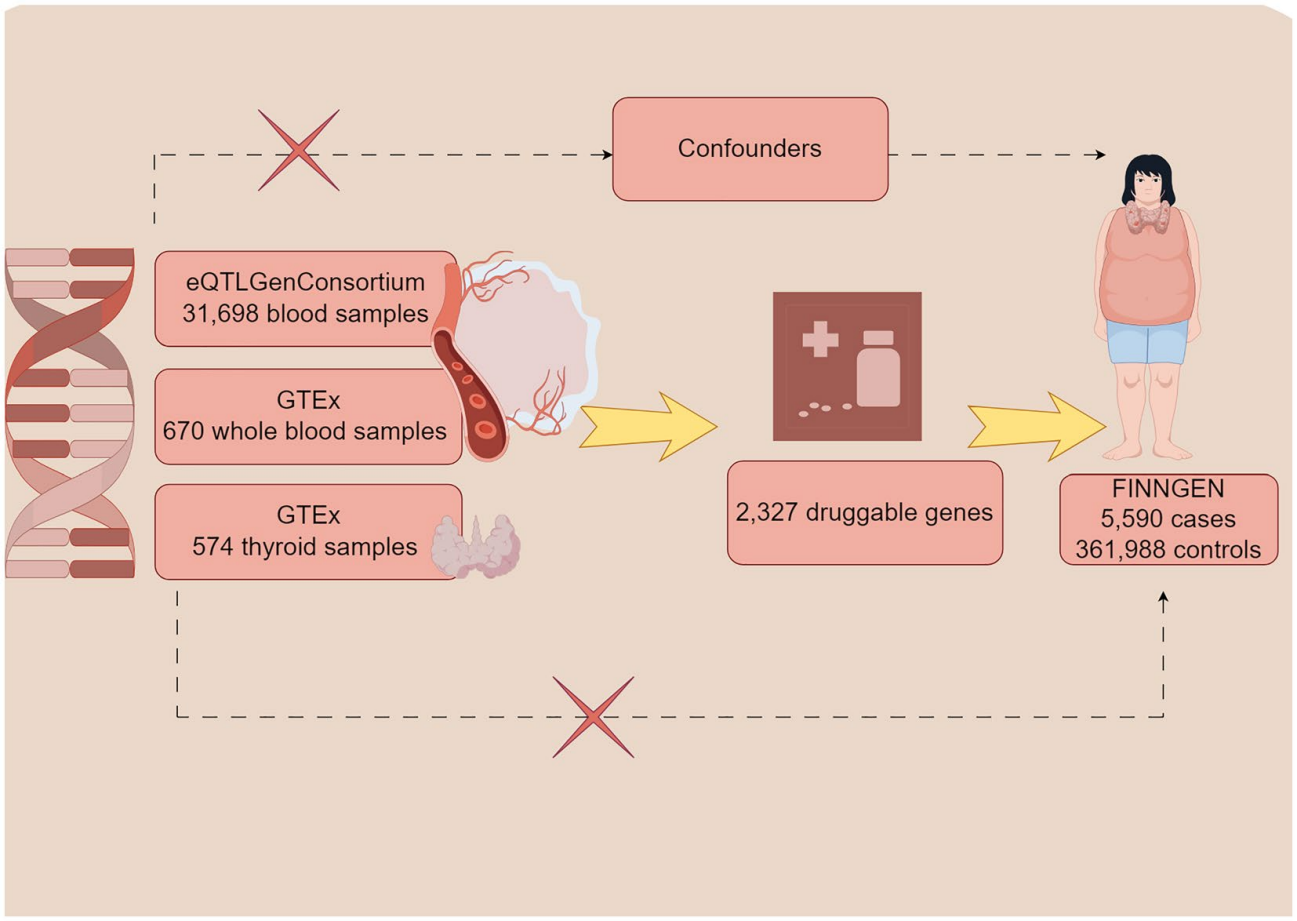
A directed acyclic graph (DAG) of this work (By Figdraw).

The present study sought to identify novel therapeutic targets relevant to hyperparathyroidism (HPT). The methodological pipeline is outlined through a flow diagram (Fig. 1) and directed acyclic graph (Fig. 2), with detailed information on data sources provided in Supplementary Table 1. First, two-sample Mendelian randomization was performed using eQTL data from eQTLGen and hyperparathyroidism GWAS from FinnGen to pinpoint potential causal genes. Second, findings were further validated via heterogeneity testing, horizontal pleiotropy assessment, and reverse causality detection. Third, analysis was replicated in GTEx data as external validation. Then, multivariable MR analysis was conducted to delineate joint causal effects of multiple risk factors. Finally, Bayesian colocalization analysis, PheWAS studies, and drug prediction were employed to investigate the feasibility of the identified drug targets for potential future clinical applications.

Our study was a secondary analysis of publicly available data. Specifically, summary-level data were obtained from three large-scale genome-wide association resources: the eQTLGenConsortium (https://eqtlgen.org/)^16^, GTEx dataset (https://gtexportal.org/home/eqtlDashboardPage)^17^, and FinnGen (https://www.finngen.fi/en)^18^. The included studies had received ethical approval from their respective institutional review boards. The current study was performed following the current guideline and the STROBE-MR checklist has been attached as supplemental material^19^.

### Actionable drug targets

A total of 4464 druggable genes located on autosomal chromosomes with HGNC annotations were identified for analysis. This set encompassed 1425 genes encoding protein targets in current clinical development, 674 genes linked to proteins engaged by approved drugs or compounds, and 2360 genes belonging to established drug target families. The study carried out by Chris et al. provides further detailed information on these feasible pharmacological targets^20^.

### eQTL data for the identification of genetic instrumental variables

Genetic instrumental variables were derived from two distinct eQTL datasets. The discovery cohort dataset utilized for this purpose comprised eQTL data sourced from the eQTLGen Consortium. Additionally, the replication cohort dataset was obtained from GTEx, ensuring a robust validation of our findings across independent datasets.

#### eQTLGen consortium

Genome-wide significant cis-eQTLs (false discovery rate < 0.05) within ± 1 Mb of probe locations were identified. The dataset utilized for this analysis, eQTLGen, encompasses 16,987 genes and was derived from the analysis of 31,684 blood samples, primarily collected from individuals of European ancestry and characterized by their overall good health. Emphasizing the relevance of eQTLs in drug development studies due to their proximity to the target genes and direct impact on gene expression, we narrowed down our selection to SNPs situated within 100 kb upstream of transcription start sites or 100 kb downstream of transcription end sites of druggable genes. Consequently,we identified eQTLs associated with 2327 druggable genes.

#### GTEx

As independent replication cohorts, eQTL data for 670 whole blood 574 and thyroid tissue was obtained from version 8 of the GTEx database (https://gtexportal.org/home/)^21^.

### Outcome data

#### Hyperparathyroidism

The GWAS data for hyperparathyroidism were sourced from FinnGen Release 8 (https://www.finngen.fi/en), which was published in December 2022. Hyperparathyroidism in FinnGen was defined based on the International Classification of Disease (ICD), encompassing 5,590 cases (ICD-8(2520), ICD-9(2520), ICD-10(550)), and 361,988 controls.

#### Risk factors

We identified 12 hyperparathyroidism risk factors encompassing chronic kidney disease^22^, chronic tubulointerstitial nephritis^18^, glomerulonephritis^18^, IgA nephropathy^18^, nephrolithiasis^18^, blood calcium levels^23^, serum/plasma magnesium^24^, serum/plasma 25-hydroxyvitamin D2/D3^24^, serum phosphate levels^25^, parathyroid hormone^26^, intestinal malabsorption^18^, and osteomalacia^18^ (Supplementary Table S2).

### Mendelian randomization analysis

The TwoSampleMR R package (version 0.5.7, https://mrcieu.github.io/ TwoSampleMR/) was employed to execute two-sample MR analysis^27^. Stringent quality control of the SNP instruments was implemented prior to MR testing. First, variants with weak instrument strength (F-statistic < 10, where F = (beta/se)^2^) were excluded. Conditionally independent variants in low linkage disequilibrium (LD r^2^ < 0.1 per 1000 Genomes European panel) were then selected. Finally, Steiger filtering removed genes wherein SNPs explained greater outcome than exposure variance (Supplementary Table S3).

In the primary analysis, the Wald ratio was employed for proposed instruments comprising a single SNP. For proposed instruments that encompassed more than one SNP, a comprehensive approach was adopted. This included the use of the inverse variance weighted (IVW) method, MR-Egger, and weighted median MR. The IVW method assumes all genetic instruments are valid and has the highest statistical power when this assumption holds ^28^. The weighted median approach allows some (50%) invalid instruments by weighting SNP-specific MR estimates based on magnitude and taking the median estimate with bootstrapped standard errors^29^. MR-Egger allows for horizontal pleiotropy, wherein some SNPs influence the outcome via alternative pathways, but has lower statistical power. The MR-Egger intercept test can determine the presence of horizontal pleiotropy^30^. To account for multiple testing in the sensitivity analyses, Bonferroni corrections were implemented to establish adjusted significance thresholds. In the eQTLGen cohort, *p* values below 2.15e−05 (calculated as *p* = 0.05/2327, 2327 is the number of druggable genes in eQTLGen data) were deemed significant. Following this, quality control procedures were applied to genes identified as significant, ensuring consistency in the direction of estimated effects across the three methods and confirming the absence of horizontal pleiotropy via the MR-Egger test. Genes passing quality control were further evaluated in the GTEx cohort. A conservative significance threshold of *P* < 1.25e−02 (calculated as 0.05/4 for Bonferroni correction, 4 is the number of genes replicated in GTEx data) was applied. Replication at this stringent cutoff enhanced robustness of associations by reducing likelihood of false positives, providing external validation complementing the original eQTLGen discovery analysis.

Additionally, multivariable MR analysis was conducted using the MVMR R package, examining associations of PIK3C3 and SLC40A1 with hyperparathyroidism risk while adjusting for the other variable. As an extension of univariate MR, multivariable MR can delineate joint causal effects of multiple risk factors ^31^. For PIK3C3, the effect of intestinal malabsorption was adjusted^18^. For SLC40A1, adjustments were made for potential effects of chronic kidney disease^22^, blood calcium levels^23^, and glomerulonephritis^18^.

### Reverse causality detection

Adhering to analogous screening criteria employed for eQTLs, genetic instruments for hyperparathyroidism were meticulously chosen from the GWAS dataset of FinnGen. These instruments were then utilized in a bidirectional MR analysis to discern possible reverse causation. Effect estimates were derived using three distinct methods: MR-Inverse Variance Weighted (MR-IVW), MR-Egger, and weighted median. Furthermore, a Steiger filtering procedure was executed to confirm the directionality of the association between expression quantitative trait loci (eQTL) and hyperparathyroidism^32^. Statistical significance was established at a threshold of *P* < 0.05, providing a rigorous criterion for the validity and reliability of the observed associations.

### Colocalization analysis

For genes with significant Mendelian randomization associations in both eQTLGen and GTEx cohorts, colocalization analysis was performed using the coloc R package with default priors ^33^. This Bayesian approach tested whether the gene expression-hyperparathyroidism links were driven by shared causal variants at a given locus rather than linkage disequilibrium. Specifically, five mutually exclusive hypotheses were assessed: (H0) no association with either trait; (H1) association with expression only; (H2) association with disease only; (H3) association with both traits, but independent causal variants; (H4) association with both traits driven by a shared causal variant^34^. Posterior probabilities were furnished for each hypothesis. Prior probabilities were set at 1e−04 for trait 1 only (p1) and trait 2 only (p2), and 1e−05 for both traits (p12). Strong evidence of colocalization was defined as a posterior probability ≥ 0.8 for H4, with values between 0.5 and 0.8 suggesting moderate colocalization.

### Phenome‑wide association analysis

Utilizing the AstraZeneca PheWAS Portal (https://azphewas.com/) and PheWeb database (https://pheweb.org/), PheWAS was carried out to thoroughly evaluate the horizontal pleiotropy of possible therapeutic targets and probable adverse effects^35,36^.

### Candidate drug prediction

In the course of this study, the identified target genes are submitted to the Drug Signatures Database (DSigDB, http://dsigdb.tanlab.org/DSigDBv1.0/) for the assessment of protein-drug interactions. DSigDB stands as a significant repository, housing 22,527 gene sets and 17,389 distinct compounds, associated with 19,531 genes. This comprehensive database facilitates the establishment of links between pharmaceuticals, diverse chemicals, and their respective target genes. PIK3C3 and SLC40A1 were analyzed using the DSigDB drug database on Enrichr (https://maayanlab.cloud/modEnrichr/) to identify potential targeting agents^37^.

## Results

### MR analysis identifies 5 actionable therapeutic targets for hyperparathyroidism

**Figure 3:**
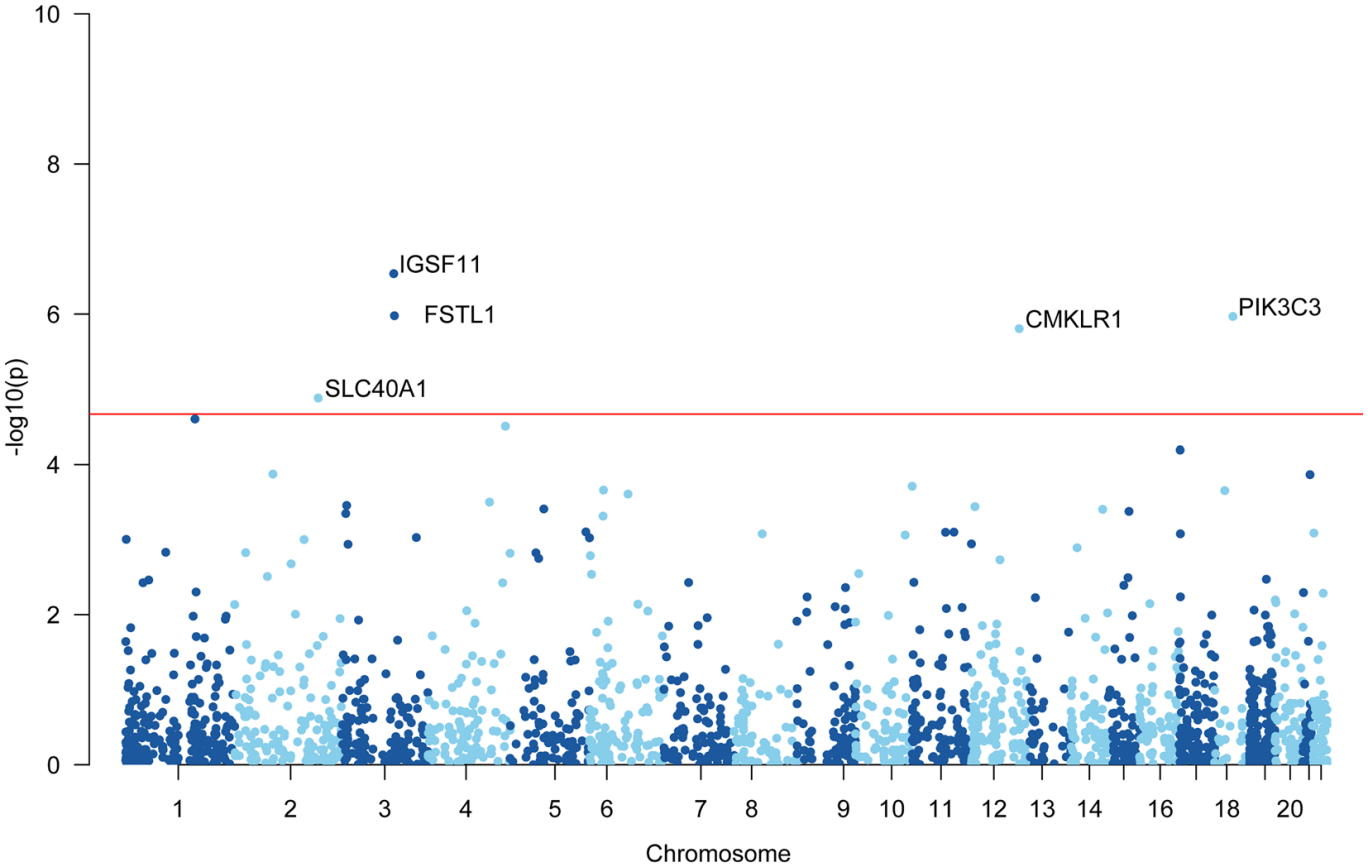
Manhattan plots for associations of genetically predicted 2327 druggable genes levels with hyperparathyroidism in MR analysis.

**Figure 4:**
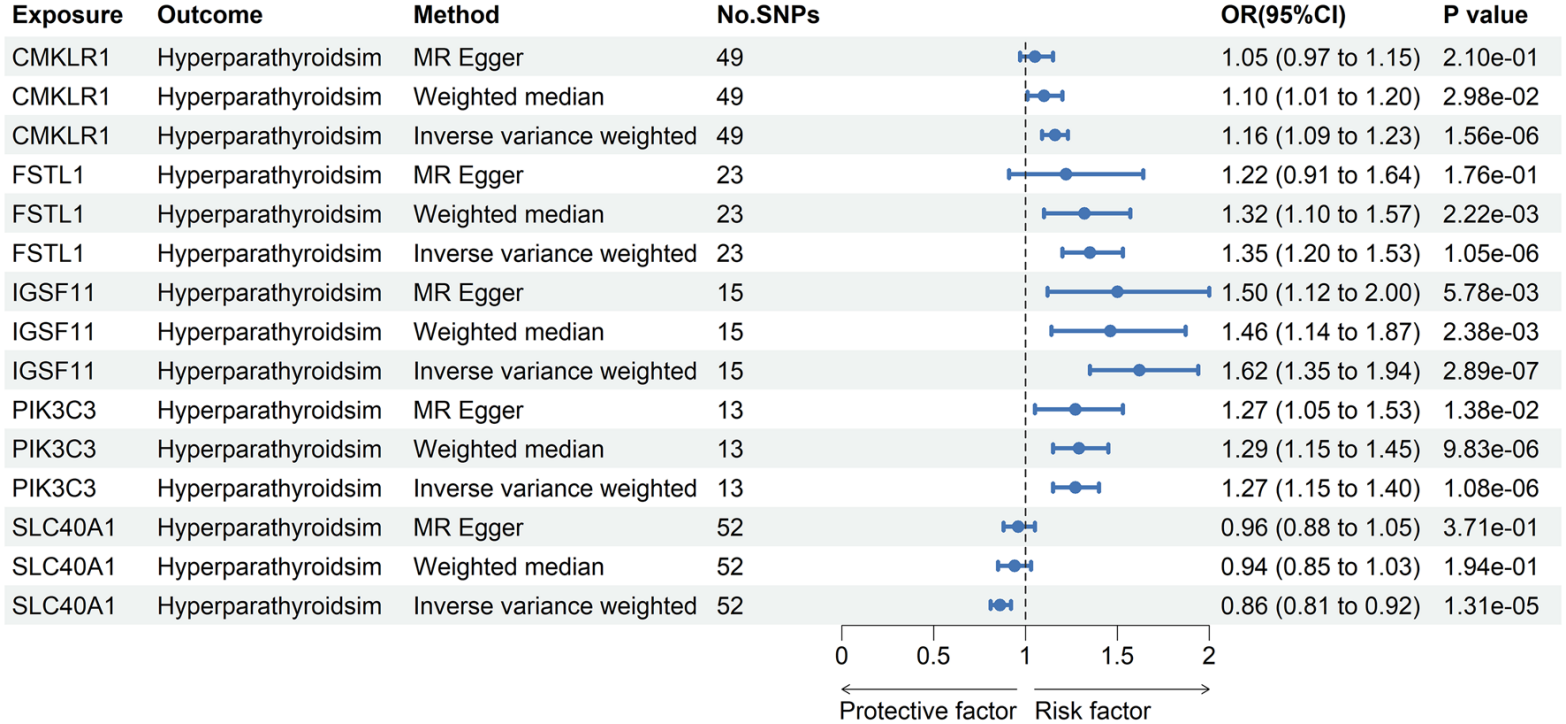
Forest plots illustrating the results of the discovery stage for 5 essential genes.

At Bonferroni significance (*P* < 2.15e−05), the MR analysis revealed that five genes are causally related to the risk of hyperparathyroidism, as illustrated in Figs. 3 and 4. These genes include chemerin chemokine-like receptor 1 (CMKLR1), follistatin like 1 (FSTL1), immunoglobulin superfamily member 11 (IGSF11), phosphatidylinositol 3-kinase catalytic subunit type 3 (PIK3C3), and solute carrier family 40 member 1 (SLC40A1). Specifically, increased expression of CMKLR1 (OR = 1.16; 95% CI 1.09–1.23; *P* = 1.56e−06), FSTL1 (OR = 1.35; 95% CI 1.2–1.53; *P* = 1.05e−06), IGSF11 (OR = 1.62; 95% CI 1.35–1.94; *P* = 2.89e−07), and PIK3C3 (OR = 1.27; 95% CI 1.15–1.4; *P* = 1.08e−06) increased the risk of hyperparathyroidism, whereas elevated SLC40A1 (OR = 0.86; 95% CI 0.81–0.92; *P* = 1.31e−05) decreased the risk of hyperparathyroidism. All 5 genes showed consistent direction of effect across the three methods (Supplemental Table S4) and no heterogeneity (*P* > 0.05, Supplemental Table S5) and horizontal pleiotropy (*P* > 0.05, Supplemental Table S6) was detected in the primary analysis. Bidirectional Mendelian randomization analysis showed no evidence for causal effects of hyperparathyroidism on expression of the five identified genes. Application of Steiger filtering provided additional confirmation of directionality from gene expression to disease status (Supplementary Tables S7 and S8).

### Replication phase 5 genes remain significant in GTEx eQTL database

**Figure 5:**
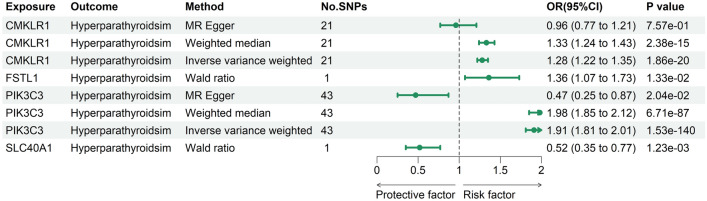
Forest plots illustrating the results of the replication stage for 4 essential genes.

As IGSF11 was not available in GTEx whole blood samples, it was excluded from subsequent replication analyses. The replication MR analysis employed identical methodology as the original discovery cohort. Genetically predicted expression of CMKLR1, PIK3C3, and SLC40A1 retained significant causal associations with hyperparathyroidism risk in GTEx whole blood samples (*P* < 1.25e−02, inverse-variance weighted methods) (Fig. 5; Supplementary Table S9). Importantly, the directionality of effect was consistent across both the discovery and replication sets for all 3 genes.

### Insights from thyroid tissue analysis

**Figure 6:**
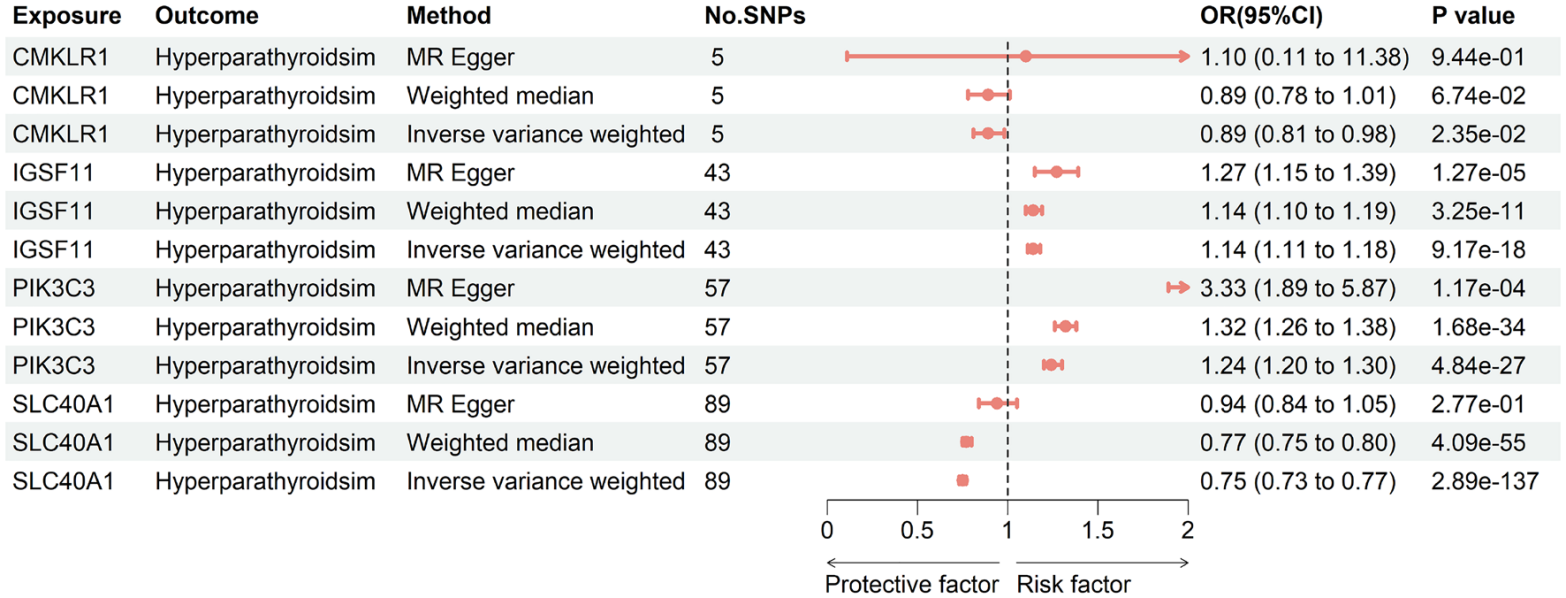
Forest plots illustrating the results of the replication stage for 4 essential genes in thyroid tissue of the GTEx V8 dataset.

The FSTL1 was not found in thyroid tissue from the GTEx V8 dataset, precluding its inclusion in replication analyses. Using an identical Mendelian randomization approach as the original discovery cohort, we examined the causal relationship between genetically predicted expression levels of several genes in the GTEx thyroid tissue cohort and hyperparathyroidism risk. Genetically predicted expression of IGSF11, PIK3C3, and SLC40A1 retained significant associations with hyperparathyroidism risk in GTEx thyroid tissue cohort (*P* < 1.25e−02, inverse-variance weighted methods) (Fig. 6; Supplementary Table S10). Importantly, the directionality of effect was consistent between the whole blood samples and thyroid tissue for all three genes.

### Colocalization analysis

**Figure 7:**
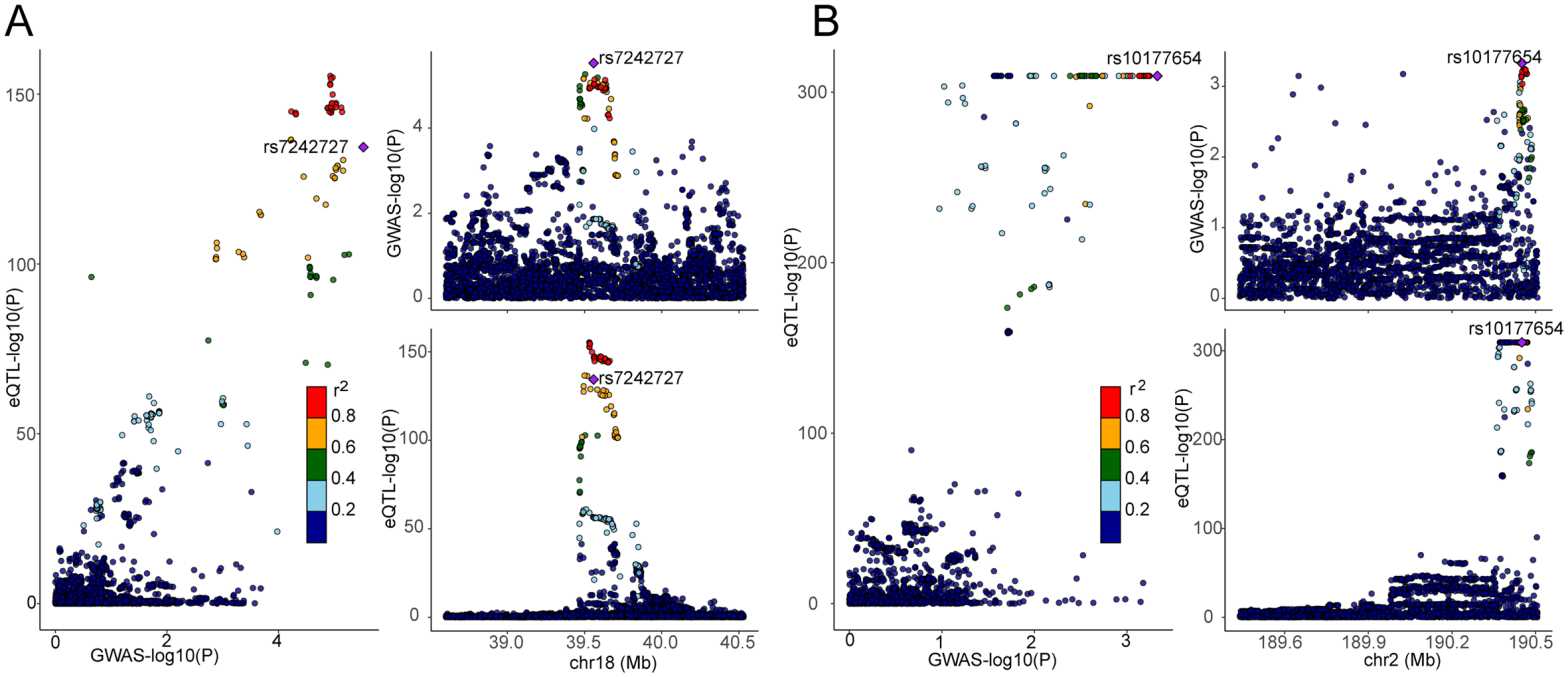
(**A**) Regional plot of colocalization evidence of PIK3C3 and hyperparathyroidism susceptibility. (**B**) Regional plot of colocalization evidence of SLC40A1 and hyperparathyroidism susceptibility.

The MR results for PIK3C3 and SLC40A1 consistently demonstrated positive or negative estimated effects in both the discovery and two testing cohorts. This consistency suggests a correlation between elevated expression of PIK3C3 and decreased expression of SLC40A1 with an increased risk of hyperparathyroidism. Consequently, antagonists of PIK3C3 and protagonists of SLC40A1, may represent a novel and robust strategy for reducing the risk of hyperparathyroidism. Prior studies have noted that significant MR findings can arise from loci where SNPs in linkage disequilibrium have associations with exposure and outcome mediated through distinct causal variants, conferring false positive results^38^. Colocalization analysis can elucidate whether exposure and outcome share the same causal SNP when SNPs demonstrate associations with both traits^33^. Bayesian co-localization strongly suggested that PIK3C3 (coloc.abf-PPH4 = 0.93) and SLC40A1 (coloc.abf-PPH4 = 0.58) shared the same variant with hyperparathyroidism , indicating a higher likelihood that these genes may serve as promising drug targets and have an increased probability of obtaining regulatory approval (Fig. 7, Supplemental Table S11).

### Multivariable MR analysis identified PIK3C3 and SLC40A1 were independent associations with hyperparathyroidism

**Figure 8:**
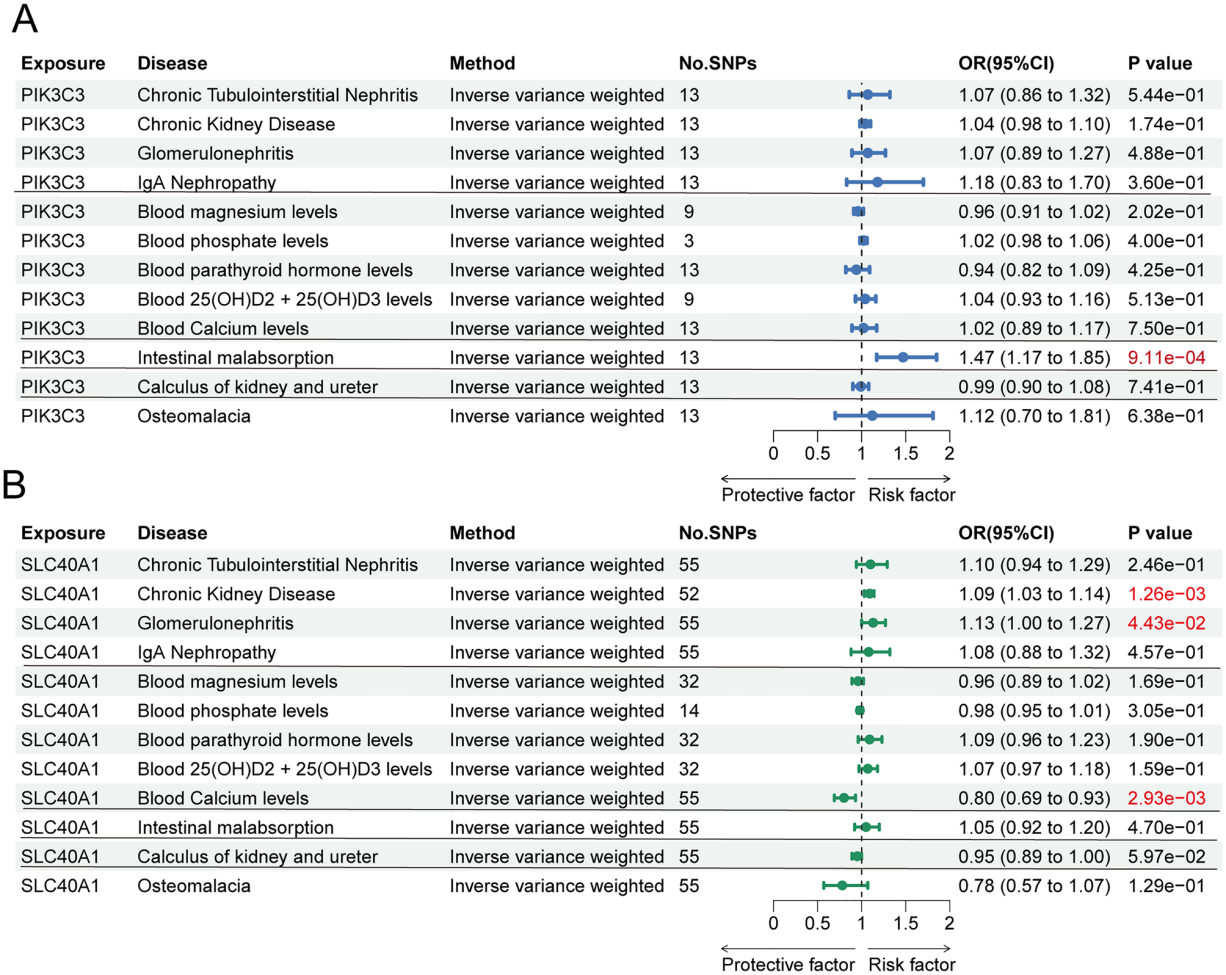
Associations between two drug targets and hyperparathyroidism risk factors. (**A**) Forest plot revealed that intestinal malabsorption is linked to the genetically predicted expression of PIK3C3. (**B**) Forest plot revealed that chronic kidney disease, glomerulonephritis and blood calcium levels is linked to the genetically predicted expression of SLC40A1.

Among the 12 hyperparathyroidism risk factors, PIK3C3 exhibited significant associations with intestinal malabsorption (*P* = 9.11e−04 [IVW], Fig. 8A). To examine the potential independent relationship between PIK3C3 and hyperparathyroidism, multivariable MR analysis was conducted, revealing significant independent associations (*P* = 2.7e−10 [IVW], Supplemental Table S12). Additionally, SLC40A1 showed weak links to chronic kidney disease (*P* = 1.26e−03), glomerulonephritis (*P* = 4.43e−02) and blood calcium levels (*P* = 2.93e−03) (Fig. 8B). Multivariable MR analysis adjusting for these potential confounders still detected a strong association between SLC40A1 and hyperparathyroidism (*P* = 6.8e−03[IVW], Supplemental Table S13).

### PheWAS

We conducted phenome-wide MR using the PheWAS Portal and PheWeb database^35,36^, in order to investigate the possible adverse consequences of these two previous druggable genes. Neither of the two drug targets showed significant associations with other traits at the gene level (*P* < 5e−08 for genomic association) in PheWAS Portal (Supplemental Figures S1–S2) and PheWeb database (Supplementary Table S14–15). This further supports the validity of the study results by indicating that the likelihood of adverse medication reactions against these targets and the existence of horizontal pleiotropy in these genes are probably low.

### Candidate drug prediction

Evaluating protein-drug interactions is critical for determining the viability of utilizing target genes as potential drug targets. PIK3C3 and SLC40A1 were analyzed using the DSigDB drug database on Enrichr to identify potential targeting agents. The findings indicate that emodin (CTD 00005893), celecoxib (CTD 00003448), and temozolomide (CTD 00002088) are the most significant drugs linked to PIK3C3, while Panobinostat (CTD 00004412), folic acid (CTD 00005997), and ethanol (CTD 00005337) are the most significant drugs linked to SLC40A1(Table 1).

**Table 1:** Candidate drug predicted using DSigDB.

Term	*P* value	Adjusted *P* value	Odds ratio	Combined score	Genes
Panobinostat	0.008	0.089	265.640	1296.720	SLC40A1
Folic acid	0.019	0.089	102.617	405.061	SLC40A1
Ethanol	0.023	0.095	85.948	324.341	SLC40A1
Emodin	0.011	0.089	179.162	805.268	PIK3C3
Celecoxib	0.012	0.089	172.896	771.052	PIK3C3
Temozolomide	0.020	0.089	97.512	380.036	PIK3C3

## Discussion

Hyperparathyroidism significantly impacts the quality of life and survival time of patients^39^. Currently, conventional drugs and surgical methods fall short of fully meeting clinical treatment needs. The development of effective and minimally invasive treatment methods is extremely necessary^40^. Thus far, this study represents the first application of MR leveraging eQTL data to systematically identify potentially molecular targets that could potentially provide protection against hyperparathyroidism.

This work identified two drug targets for hyperparathyroidism: PIK3C3 and SLC40A1 employing a variety of MR methods (MR-Egger, Wald ratio/IVW, weighted median method, Cochran’s Q heterogeneity test, horizontal multiplicity test, bidirectional MR analysis, Steiger test, colocalization analysis and multivariable MR analysis). Moreover, the association between PIK3C3 and SLC40A1 with hyperparathyroidism was corroborated using a parallel analysis in the GTEx database, reinforcing the credibility of the potential drug targets identified in this investigation. To further elucidate potential pleiotropy and off-target effects, PheWAS analysis was also conducted. Ultimately, the study also showed the druggable value of these target genes by predicting compounds matching to these targets.

PIK3C3, also known as VPS34, is a member the family of phosphoinositide 3-kinases—lipid kinases that play a key role in autophagy^41^. There are several consecutive stages that make up the autophagy machinery: initiation, nucleation, elongation, fusion, and degradation^42^. Specifically, PIK3C3/VPS34 interacts with BECN1/Beclin 1 to form a complex central to autophagosome nucleation^41^. Previous studies have demonstrated that autophagy inhibitors can potentiate effects of 5-aminolevulinic acid-mediated photodynamic therapy in secondary hyperparathyroidism models^40^. Additionally, calcimimetics that activate calcium-sensing receptors in parathyroid tissue have been utilized to clinically manage hyperparathyroidism, where they are proposed to ameliorate deleterious tissue remodeling in part by modulating autophagy^43,44^. Intriguingly, our analyses newly identify genotype–phenotype associations between PIK3C3 variants and altered hyperparathyroidism risk (eQTLGen odds ratio OR = 1.27, *P* = 1.08e−06; GTEx whole blood samples OR = 1.91, *P* = 1.53e−140; GTEx thyroid tissue OR = 1.25, *P* = 4.84e−27), strongly supported by colocalization (PPH4 = 0.933). Moreover, phenome-wide and multivariate Mendelian randomization indicate effects likely represent direct modulation of hyperparathyroidism pathology rather than widespread pleiotropy. These results suggest that the PIK3C3 inhibition as a promising hyperparathyroidism therapeutic strategy worthy of future investigation.

The protein encoded by SLC40A1 functions as a cell membrane protein, potentially facilitating the transport of Fe (2 +) from the interior to the exterior of the duodenal epithelial cells^45,46^. A portion of ferric iron in the duodenum binds to phosphorus, forming an insoluble compound that is subsequently excreted in feces^47^. This process contributes to the reducing of serum phosphorus, thereby inhibiting parathyroid hormone secretion. Our MR analysis suggested that SLC40A1, a mediator of Fe translating, may be a promising hyperparathyroidism therapeutic targets (eQTLGen odds ratio OR = 0.86, *P* = 1.31e−05; GTEx whole blood samples OR = 0.52, *P* = 1.23e−03; GTEx thyroid tissue OR = 0.75, *P* = 2.89e−137; PPH4 = 0.581).

Pharmacological treatments, including calcium and calcium-binding agents (calcium acetate and carbonic acid), non-calcium-binding agents (velamum and lanthanum carbonate), vitamin D and its analogs, and pseudocalcifier (sikansei), have advanced recently due to significant advancements in the molecular mechanisms of HPT. In addition, although parathyroidectomy (PTX) is effective in partly SHPT patients, the tolerance of patients with SHPT is poor and the incidence risk of perioperative period is still high. Given the side effects of drugs, inconclusive clinical trial outcomes, and the limitations of surgery, it becomes imperative to explore alternative therapeutic targets that are potentially effective and safe. Thus, to ensure the credibility of results derived from the MR analysis, we employed gene expression as the exposure and disease summary statistics from the UK Biobank cohorts to perform phenome-wide MR based on PheWAS Portal and PheWeb database. The findings indicate that the potential side effects of drugs are minimal and substantially reducing the likelihood of biased results attributable to pleiotropy.

This study has several strengths, including an extensive collection of eQTL, a substantial number of hyperparathyroidism cases, mutual validation in two independent exposure datasets, supportive colocalization analysis, and the exclusion of horizontal pleiotropy through PheWAS and multivariate MR analyses. Limitations should be considered when interpreting our findings. Firstly, our analyses were restricted to individuals of European descent, which limits the generalizability of the results to other ethnic populations. Secondly, due to the paucity of genetic studies on protein levels, we did not identify protein quantitative trait loci (pQTL) for PIK3C3 and SLC40A1. Consequently, we were unable to validate whether protein levels of PIK3C3 and SLC40A1 consistently associated with hyperparathyroidism risk. Additionally, clinical trials are imperative to evaluate the efficacy and safety of targeting PIK3C3 or SLC40A1 for therapeutic management of hyperparathyroidism. Furthermore, while Mendelian randomization provides insight into potential causal relationships, it assumes low-dose chronic exposure and linear dose–response links that may not fully extrapolate to real-world clinical trials that often feature short-term high-dose treatments. Consequently, effect sizes estimated in our analyses may not precisely mirror those observed under standard pharmacological parameters. Finally, owing to the lack of additional GWAS datasets for hyperparathyroidism itself, we could only verify conclusions using independent exposure but not outcome data.

In summary, this cis-eQTL-wide Mendelian randomization and colocalization analysis identified PIK3C3 inhibitors and SLC40A1 antagonists as promising therapeutic targets for hyperparathyroidism. Nevertheless, randomized controlled trials are imperative to conclusively assess the efficacy and safety of these potential drug targets.

### Supplementary Information

Supplementary Information 1.

Supplementary Information 2.

Supplementary Information 3.

## Data Availability

The GWAS datasets generated and/or analyzed during the current study are publicly available.

## References

[1] Huimin C (2018). Effects of parathyroidectomy on plasma iPTH and (1–84) PTH levels in patients with stage 5 chronic kidney disease. Horm. Metab. Res..

[2] Kono K, Fujii H, Watanabe K, Goto S, Nishi S (2021). Relationship between parathyroid hormone and renin-angiotensin-aldosterone system in hemodialysis patients with secondary hyperparathyroidism. J. Bone Miner. Metab..

[3] Ishida H (2020). Skeletal and mineral metabolic effects of risedronate in a rat model of high-turnover renal osteodystrophy. J. Bone Miner. Metab..

[4] Cappellacci F (2020). Parathyroid carcinoma in the setting of tertiary hyperparathyroidism: Case report and review of the literature. Case Rep. Endocrinol..

[5] Rodrigo JP (2020). Parathyroid cancer: An update. Cancer Treat. Rev..

[6] Bilezikian JP (2022). The fifth international workshop on the evaluation and management of primary hyperparathyroidism. J. Bone Miner. Res..

[7] Zhang LX (2022). Advances in the treatment of secondary and tertiary hyperparathyroidism. Front. Endocrinol. Lausanne.

[8] Fraser WD (2009). Hyperparathyroidism. Lancet.

[9] Reay WR, Cairns MJ (2021). Advancing the use of genome-wide association studies for drug repurposing. Nat. Rev. Genet..

[10] Schmidt AF (2020). Genetic drug target validation using Mendelian randomisation. Nat. Commun..

[11] Zhu Z (2016). Integration of summary data from GWAS and eQTL studies predicts complex trait gene targets. Nat. Genet..

[12] Chen Y (2022). Genetic insights into therapeutic targets for aortic aneurysms: A Mendelian randomization study. EBioMedicine.

[13] Gaziano L (2021). Actionable druggable genome-wide Mendelian randomization identifies repurposing opportunities for COVID-19. Nat. Med..

[14] Cao Y, Yang Y, Hu Q, Wei G (2023). Identification of potential drug targets for rheumatoid arthritis from genetic insights: a Mendelian randomization study. J. Transl. Med..

[15] Storm CS (2021). Finding genetically-supported drug targets for Parkinson's disease using Mendelian randomization of the druggable genome. Nat. Commun..

[16] Võsa U (2021). Large-scale cis- and trans-eQTL analyses identify thousands of genetic loci and polygenic scores that regulate blood gene expression. Nat. Genet..

[17] Human genomics. The Genotype-Tissue Expression (GTEx) pilot analysis: Multitissue gene regulation in humans. *Science* **348**, 648–660. 10.1126/science.1262110 (2015). 10.1126/science.1262110 PMC4547484 25954001

[18] Kurki MI (2023). FinnGen provides genetic insights from a well-phenotyped isolated population. Nature.

[19] Skrivankova VW (2021). Strengthening the reporting of observational studies in epidemiology using Mendelian randomisation (STROBE-MR): Explanation and elaboration. BMJ.

[20] Finan C (2017). The druggable genome and support for target identification and validation in drug development. Sci. Transl. Med..

[21] The GTEx Consortium atlas of genetic regulatory effects across human tissues. *Science* **369**, 1318–1330. 10.1126/science.aaz1776 (2020). 10.1126/science.aaz1776 PMC7737656 32913098

[22] Pattaro C (2016). Genetic associations at 53 loci highlight cell types and biological pathways relevant for kidney function. Nat. Commun..

[23] Rusk N (2018). The UK biobank. Nat. Methods.

[24] Dennis JK (2021). Clinical laboratory test-wide association scan of polygenic scores identifies biomarkers of complex disease. Genome Med..

[25] Sinnott-Armstrong N (2021). Genetics of 35 blood and urine biomarkers in the UK Biobank. Nat. Genet..

[26] Sun BB (2018). Genomic atlas of the human plasma proteome. Nature.

[27] Hemani G (2018). The MR-Base platform supports systematic causal inference across the human phenome. Elife.

[28] Burgess S (2019). Guidelines for performing Mendelian randomization investigations: Update for summer 2023. Wellcome Open Res..

[29] Bowden J, Davey Smith G, Haycock PC, Burgess S (2016). Consistent estimation in mendelian randomization with some invalid instruments using a weighted median estimator. Genet. Epidemiol..

[30] Bowden J, Davey Smith G, Burgess S (2015). Mendelian randomization with invalid instruments: effect estimation and bias detection through Egger regression. Int. J. Epidemiol..

[31] Sanderson E, DaveySmith G, Windmeijer F, Bowden J (2019). An examination of multivariable Mendelian randomization in the single-sample and two-sample summary data settings. Int. J. Epidemiol..

[32] Hemani G, Tilling K, Davey Smith G (2017). Orienting the causal relationship between imprecisely measured traits using GWAS summary data. PLoS Genet..

[33] Giambartolomei C (2014). Bayesian test for colocalisation between pairs of genetic association studies using summary statistics. PLoS Genet..

[34] Foley CN (2021). A fast and efficient colocalization algorithm for identifying shared genetic risk factors across multiple traits. Nat. Commun..

[35] Wang Q (2021). Rare variant contribution to human disease in 281,104 UK Biobank exomes. Nature.

[36] Gagliano Taliun SA (2020). Exploring and visualizing large-scale genetic associations by using PheWeb. Nat. Genet..

[37] Kuleshov MV (2016). Enrichr: a comprehensive gene set enrichment analysis web server 2016 update. Nucleic Acids Res..

[38] Hemani G, Bowden J, Davey Smith G (2018). Evaluating the potential role of pleiotropy in Mendelian randomization studies. Hum. Mol. Genet..

[39] Bilezikian JP, Bandeira L, Khan A, Cusano NE (2018). Hyperparathyroidism. Lancet.

[40] Zeng L (2021). Inhibition of autophagy with Chloroquine enhanced apoptosis induced by 5-aminolevulinic acid-photodynamic therapy in secondary hyperparathyroidism primary cells and organoids. Biomed. Pharmacother..

[41] Axe EL (2008). Autophagosome formation from membrane compartments enriched in phosphatidylinositol 3-phosphate and dynamically connected to the endoplasmic reticulum. J. Cell Biol..

[42] Yang G, Driver JP, Van Kaer L (2018). The role of autophagy in iNKT cell development. Front. Immunol..

[43] Nemeth EF (2013). Allosteric modulators of the extracellular calcium receptor. Drug Discov. Today Technol..

[44] Liu L (2016). Suppression of calcium-sensing receptor ameliorates cardiac hypertrophy through inhibition of autophagy. Mol. Med. Rep..

[45] Schimanski LM (2005). In vitro functional analysis of human ferroportin (FPN) and hemochromatosis-associated FPN mutations. Blood.

[46] Zhang DL (2018). Erythrocytic ferroportin reduces intracellular iron accumulation, hemolysis, and malaria risk. Science.

[47] Ganz T, Bino A, Salusky IB (2019). Mechanism of action and clinical attributes of Auryxia(®) (ferric citrate). Drugs.

