# Supplementary material for: Unveiling potential drug targets for hyperparathyroidism through genetic insights via Mendelian randomization and colocalization analyses
Source: Sci Rep. 2024 Mar 18;14:6435. doi: 10.1038/s41598-024-57100-3 (PMC10948885; doi:10.1038/s41598-024-57100-3)
Supplement: Supplementary file 2 — Supplementary Information 2. [file 41598_2024_57100_MOESM2_ESM.pdf]

Unveiling Potential Drug Targets for Hyperparathyroidism through Genetic Insights via Mendelian Randomization and Colocalization Analyses

Bohong Chen [1], Lihui Wang [2], Shengyu Pu [3], Li Guo[1], Na Chai [3], Xinyue Sun[4], Xiaojiang Tang [3], Yu Ren [3], Jianjun He[3*], Na Hao [3*]

1. Department of Urology, the First Affiliated Hospital of Xi’an Jiaotong University, Xi’an, 710061, Shaan’xi Province, China.
2. Department of Obstetrics and Gynecology, the First Affiliated Hospital of Xi’an Jiaotong University, Xi’an, 710061, Shaan’xi Province, China.
3. Department of Breast Surgery, the First Affiliated Hospital of Xi’an Jiaotong University, Xi’an, 710061, Shaan’xi Province, China.
4. Department of neurology, The First Affiliated Hospital of Xi'an Jiaotong University, 710061 Xi'an, Shaan’xi, China.

*Corresponding author: Na Hao, Ph.D., M.D., associate professor in Department of Breast Surgery, First Affiliated Hospital, School of Medicine, Xi’an Jiaotong University. Email: haona0318@xjtufh.edu.cn; Tel: (+86) 19929906958. Address: 277 Yanta Western Rd., Xi’an 710061, Shaan’xi Province, China.

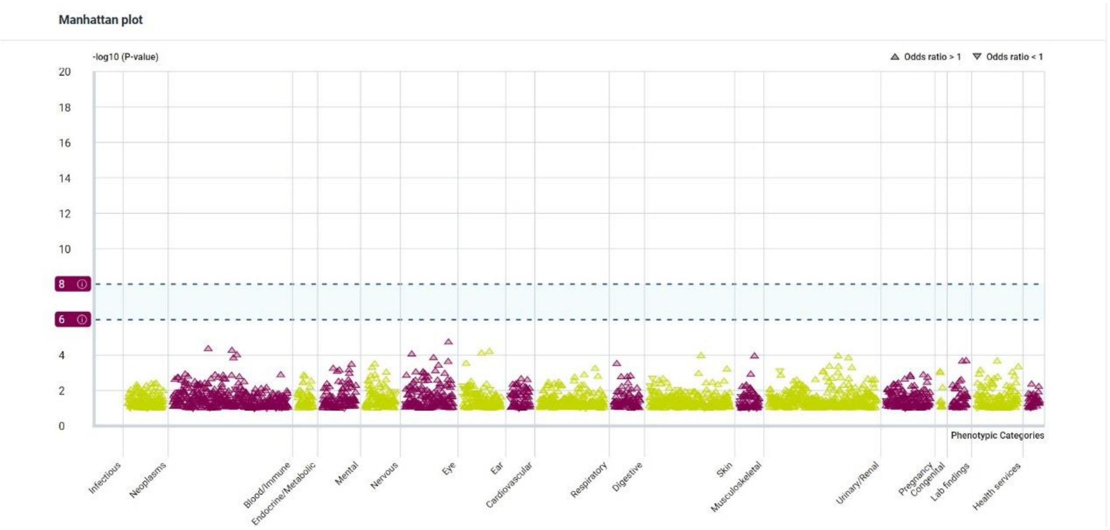

Figure S1. Binary traits PheWAS association with PIK3C3.

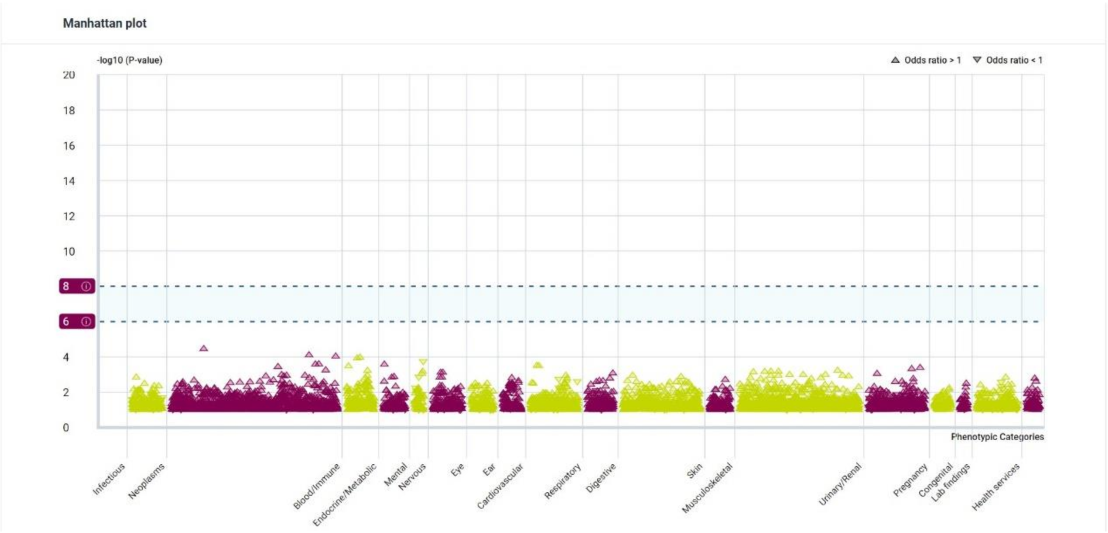

Figure S2. Binary traits PheWAS association with SLC40A1.
